# Long-Term Effectiveness of Onabotulinum Toxin-A in a Combined Total Endoscopic Management of Pediatric Vesicoureteral Reflux in Neurogenic Bladder Dysfunction

**DOI:** 10.3390/toxins17070330

**Published:** 2025-06-29

**Authors:** Claudio Paratore, Chiara Pellegrino, Noemi Deanesi, Rebecca Pulvirenti, Maria Luisa Capitanucci, Giovanni Mosiello

**Affiliations:** 1 Division of Neuro-Urology, Bambino Gesù Children’s Hospital, IRCCS, ERN eUROGEN Affiliated Center, Piazza di Sant’Onofrio 4, 00165 Rome, Italy; claudio.paratore@virgilio.it (C.P.); noemi.deanesi@unicampus.it (N.D.); rebecca.pulvirenti@opbg.net (R.P.); marialuisa.capitanucci@opbg.net (M.L.C.); giovanni.mosiello@opbg.net (G.M.); 2 Department of Neuroscience, Rehabilitation, Ophthalmology, Genetics and Maternal-Child Sciences, University of Genoa, Via Balbi 5, 16126 Genoa, Italy; 3 Department of Urology, Campus Bio-Medico University of Rome, Via Alvaro del Portillo 21, 00128 Rome, Italy

**Keywords:** vesicoureteral reflux, neurogenic bladder dysfunction, endoscopic management, minimally invasive, Onabotulinum Toxin-A, Deflux, pediatric, urinary tract infection

## Abstract

Vesicoureteral reflux (VUR) management in children with neurogenic bladder dysfunction (NBD) remains a clinical challenge. Total endoscopic management (TEM), combining intradetrusor Onabotulinum Toxin-A (BTX-A) and subureteric dextranomer/hyaluronic acid (Deflux^(R)^) injection, offers a minimally invasive alternative. The aim of this retrospective study is to evaluate the long-term effectiveness of TEM. Inclusion criteria: symptomatic II–V grade VUR (also I in bilateral VUR) in NBD children with follow-up ≥12 months. Nineteen patients were enrolled, 24 ureters (grade I–II: 2, grade III–V: 22); 5 patients (20.8%) had bilateral VUR. Mean age at surgery: 7.6 years (1.3–17). No complications were reported. TEM was effective in 11 patients (57.9%), 3/11 requiring a second TEM treatment. VUR resolution appeared in 14 ureters (58.3%), downgrading in 6 (42.9%), persistence in 4 (28.6%). Among non-responders’ patients (8/19, 42.1%), five (26.3%) required bladder augmentation (one combined with ureteral reimplantation), one (5.3%) underwent reimplantation, and two (10.5%) continued conservative management. At bladder biopsy, 11 patients (57.9%) had chronic inflammation, 8 (42.1%) showed fibrosis; no difference in success rate was recorded. All responders required repeated BTX-A injections. Mean follow-up: 3.2 years (range 1–4.7). In selected patients, TEM appears to be a safe and effective strategy, potentially delaying or avoiding major reconstructive surgery.

## 1. Introduction

The Neurogenic Bladder Dysfunction (NBD) is often associated with Vesicoureteral Reflux (VUR), a condition that can lead to urinary tract infections (UTIs) and deterioration of renal function [1].

In children, NBD is most frequently caused by spinal dysraphism, including myelomeningocele (or open spina bifida) and occult spinal dysraphism, and spinal cord injuries. Neurogenic bladder (NB) may manifest through various patterns of detrusor-sphincter dyssynergia, potentially resulting in urinary and/or fecal incontinence, VUR with recurrent UTIs and progressive renal damage, culminating in chronic kidney disease, needing dialysis or renal transplantation. About 12% of newborns with myelodysplasia exhibit no evident neuro-urological abnormalities at birth. However, bladder dysfunction typically emerges during early childhood, as a consequence of progressive degeneration of neural innervation [2,3,4,5].

As already mentioned, vesicoureteral reflux represents an important predisposing factor for the development of UTIs and renal scarring [6]. It may result either from an intrinsic defect of the ureterovesical junction (UVJ) or from functional overload of an anatomically normal UVJ, as seen in cases of bladder dysfunction due to congenital, acquired, or behavioral causes. These mechanisms are traditionally classified as primary and secondary reflux, respectively, though multiple contributing factors may coexist in individual patients, making these categories not entirely exclusive. Regardless of the etiology, the primary intents of VUR management are the prevention of UTIs and the preservation of renal function. In the context of NB, VUR is generally considered a secondary phenomenon, primarily driven by increased intravesical pressures rather than an inherent abnormality of the UVJ [7]. In children with neurogenic bladder, secondary vesicoureteral reflux may occur in as many as 30% of cases, most associated with detrusor-urethral sphincter dyssynergia and impaired bladder compliance [8].

Therefore, treatment of VUR in these patients overlaps with treatment of bladder dysfunction. First-line therapy for NBD consists of early management of bladder emptying, performing Clean Intermittent Catheterization (CIC), with the addition of anticholinergic drugs (e.g., oxybutynin) [9]. Today, the use of intra-detrusor injection of Onabotulinum Toxin-A (BTX-A) in the treatment of NBD is considered a common practice, gaining increasing popularity, even in pediatric patients. BTX-A is a commercial preparation of the neurotoxin produced by Clostridium Botulinum. This toxin is able to lead to a relaxation of smooth and striated muscle fibers through a complex mechanism, which is still not fully understood. BTX-A certainly leads to inhibition of the presynaptic release of acetylcholine, but other neurotransmitters and neurological patterns are involved; for example, BTX-A can inhibit the release of calcitonin gene-related peptide or decreas suburothelium receptors, such as TRPV1 (transient receptor potential vanilloid 1) and P2X (P2X purinoceptor) receptors, involved in inflammatory and nociceptive pathways. The therapeutic effect of BTX-A is time-limited (3–12 months); therefore, periodic injections are needed to maintain the improvement in bladder function. The need for repeated hospitalization is one of the most important factors for the abandonment of this therapy: 39.6–49.1% in adult patients. Pellegrino et al. found a major discontinuation rate (76%) in a retrospective evaluation of 54 pediatric patients with a 10-year follow-up [10,11,12].

In non-responders’ patients, meaning those with recurrent UTIs and ongoing VUR, different strategies have been suggested for VUR management: continuous antibiotic prophylaxis (CAP), minimally invasive endoscopic treatment with sub-ureteric injection of bulking agents (such as dextranomer/hyaluronic acid, Deflux^(R)^), uretero-vesical reimplantation associated or not with bladder augmentation [2,7,10].

In the past years, Total Endoscopic Management (TEM) was suggested as an alternative management of VUR in NBD patients. TEM consists of a combined endoscopic injection of BTX-A (intradetrusor injections) and Deflux^(R)^ (sub-ureteric injections) [13,14,15].

To date, there are few reports in the literature regarding the use of TEM for the treatment of VUR in pediatric patients with NBD; none of these studies present a long-term follow-up [13,14,15]. In view of this premise and the good results reported in the literature regarding this minimally invasive endoscopic approach, we decided to retrospectively evaluate and share our experience in terms of the safety and efficacy of TEM in the treatment of symptomatic VUR in these complex patients.

## 2. Results

Among the 22 patients initially considered, 19 were enrolled in the study (10 males, 9 females).

Three patients were excluded for the following reasons: two had a follow-up of less than one year, and one had previously undergone vesicoureteral reimplantation.

Patients’ demographic and clinical data are summarized in Table 1.

Fifteen patients (78.9%) were diagnosed with spina bifida (SB), including 5 with open SB and 10 with an occult form. The remaining four patients (21.1%) had other neurological conditions, such as central paralysis (2 patients), sequelae of sacrococcygeal teratoma (1 patient), and sacral arachnoid cyst (1 patient).

The mean age at surgery was 7.6 years (range: 1.3–17.7 years, SD 4.8).

A total of 24 ureters were treated, with 5 patients (20.8%) presenting bilateral VUR. According to the International Grading System [16], we found grade I in 2 ureters (8.3%), grade III in 7 (29.2%), grade IV in 12 (50%) and grade V in 3 (12.5%).

Five patients (20.8%) had been previously treated with BTX-A injections alone for a low-compliance bladder, but this therapeutic approach failed to resolve VUR.

All patients underwent bladder wall biopsies during the TEM procedure. Evidence of chronic inflammation was found in 11 cases (57.9%), while the other 8 samples (42.1%) showed signs of fibrosis; no significant differences were found in terms of treatment success between the two groups (*p* 0.67). All bilateral VUR showed chronic inflammation.

The mean operative time was 13 min (9–17 min; SD 2.4). No intraoperative or postoperative complications were observed. The mean postoperative hospital stay was one day (1–3 days; SD 0.7).

Overall, TEM was effective in 14 out of 24 evaluable ureters (58.3%). Among the remaining 10 ureters (41.7%), 6 showed VUR downgrading while 4 remained unchanged.

Considering patient-based outcomes, TEM has been shown to be effective in 11 out of 19 patients (57.9%). Of these, three patients had a second TEM procedure, with a resolution of VUR in only one of those.

All responder patients had to repeat BTX-A injections every 6 to 14 months (mean interval: 8.5 months) to maintain bladder compliance.

In eight patients, TEM failed. Due to subsequent clinical worsening, 6 patients were subjected to surgery: 2 (25%) underwent bladder augmentation with continent catheterizable channel creation (Mitrofanoff appendicovescicostomy); 2 (25%) underwent bladder augmentation alone; 1 (12.5%) underwent bladder augmentation combined with ureterovesical reimplantation; 1 (12.5%) underwent isolated ureterovesical reimplantation. Respecting the wishes of the patients and their family, and considering the stability of the clinical conditions, 2 (25%) proceeded with conservative management consisting of CIC, CAP and repeated intradetrusor BTX-A injections.

The mean duration of follow-up was 3.2 years (1–4.7 years, SD 1.1).

At the last urological evaluation, the management of responder patients included different approaches to bladder emptying. Nine patients (81.8%) were managed with urethral CIC. One patient (9.1%) underwent a Mitrofanoff appendicovescicostomy creation. One patient (9.1%) had a button cystostomy implantation [17].

The outcomes are summarized in Table 2.

## 3. Discussion

Neurogenic bladder encompasses a spectrum of Lower Urinary Tract Dysfunctions (LUTD) caused by damage to the central or peripheral nervous system. In the pediatric population, the most frequent etiologies are congenital anomalies such as neural tube defects (NTDs), including myelomeningocele, spinal dysraphism, and tethered cord syndrome [4,18]. In contrast, in adolescents and young adults, neurogenic bladder is often acquired following spinal cord injury (SCI), neoplastic conditions affecting the spine, or progressive neurological disorders such as multiple sclerosis [19,20].

Regardless of the underlying cause, NB is associated with considerable morbidity. Advances in surgical and medical care have significantly improved life expectancy in children with NTDs. Nevertheless, bladder dysfunction remains a major contributor to long-term complications and reduced quality of life. Similarly, bladder-related issues continue to affect more than 80% of SCI patients. Timely assessment and intervention are essential across all patient groups to preserve renal function and achieve continence [6].

In infants with open NTD, an early evaluation of the urinary tract is recommended soon after surgical repair of the spinal defect. While specific timelines are less defined for SCI patients, it is generally advised to complete a full neuro-urological evaluation, including radiological and urodynamic exams, within three months after the injury occurred [21]. Furthermore, some studies suggested that an earlier assessment may help to identify high-risk features before permanent damage occurs, although early findings may reflect transient dysfunction during spinal shock and require follow-up testing. Therefore, invasive surgical procedures should be deferred for at least one year after the spinal injury to evaluate if a recovery may occur [22,23].

Neurogenic LUTD (NLUTD) requires a complex and highly specialized assessment, including clinical, laboratory, radiological and urodynamic evaluation; usually, it is recommended that a multidisciplinary consultation of those children, with a pediatric urologist, neurosurgeon, intestinal surgeon, gastroenterologist, physiatrist and nurse specialized uroterapist [24,25].

Among different exams, a urodynamic study can make bladder dysfunction clearer. In pediatric patients, as in adults, urodynamic exams should measure bladder capacity and compliance, detrusor overactivity, intravesical pressure, and leak point pressure. A threshold of >40 cm H_2_O for intravesical pressure is often used to define high-risk cases [26]. Children in this category have been shown to exhibit worse renal function, higher rates of VUR, more frequent UTIs, and a greater likelihood of upper urinary tract deterioration. Furthermore, in children, the end-filling phase pressure between 25 and 40 cm H_2_O is considered a risk factor for kidney damage, requiring careful management. In pediatric patients, non-invasive procedures are initially preferred, with evaluation of bladder wall thickness and post-void residual as indicators of bladder dysfunction, opting for invasive tests if pathological elements are found [27,28,29,30].

In a systematic review, Veenboer et al. analyzed long-term outcomes in patients with spina bifida, reporting an annual mortality risk of approximately 1% between the ages of 5 and 30 years. Renal failure was identified as the leading cause of death, accounting for nearly 30% of cases [31]. These findings are particularly significant given that most individuals with SB are born with normal upper urinary tracts (UUT), yet up to 60% may experience UUT deterioration over time because of NLUTD. Timely intervention in NLUTD is crucial for preventing upper urinary tract deterioration. As in adults, even in pediatric patients, CIC combined with anticholinergic therapy remains the cornerstone of first-line management of NB. In cases where anticholinergic agents are poorly tolerated or prove ineffective, intradetrusor injection of BTX-A has emerged as a widely adopted alternative. Its use in the pediatric population has steadily increased over the past two decades [12,32].

It is essential to limit the occurrence of vesicoureteral reflux nephropathy to prevent chronic kidney damage. The underlying cause of VUR in patients with NB lies, as already mentioned, in the high bladder pressures caused by neurogenic bladder dysfunction, rather than in a primitive anatomical defect of the ureterovesical junction. Consequently, standard treatments for VUR, such as endoscopic sub-ureteric injection of bulking agents, may prove less effective if bladder dysfunction is not concurrently addressed [33].

CAP is commonly prescribed in infants presenting with VUR grade III to V [34]. The Randomized Intervention for Children with Vesicoureteral Reflux (RIVUR) trial, one of the most comprehensive studies to date, enrolled 607 children without neurogenic bladder after their first or second UTIs. Notably, over 90% of participants were female, most had grade II or III VUR, and the age distribution was broad. While CAP demonstrated efficacy in reducing the rate of recurrent UTIs, it did not show a significant impact on the incidence of renal scarring, raising concerns regarding its overall clinical benefit [35]. Similarly, the utility of CAP in infants with higher-grade VUR (III–V), particularly in those without prior UTIs but with potential congenital renal anomalies, remains uncertain. Additionally, its routine use must be balanced against the risks of developing multidrug-resistant organisms and the possible disruption of the gut microbiota, with a major impact on public health [36,37]. The use of CAP in pediatric patients with neurogenic bladder remains a matter of debate. However, a recent systematic review concluded that CAP is not recommended in children with neurogenic bladder and associated vesicoureteral reflux, due to insufficient evidence of benefit and concerns regarding antimicrobial resistance [38].

In pediatric patients with symptomatic UTIs during CAP, treatment options may involve surgical procedures, including endoscopic correction, ureteral reimplantation, or a multimodal approach [39]. The surgical strategy is primarily guided by factors such as the extent of bladder wall trabeculation, urethral pressure profiles, and overall bladder function assessed through urodynamic studies [7,40,41]. Different surgical techniques, both intravesical and extravesical, have been developed to correct VUR. While each approach has its own specific advantages and potential complications, they are all based on the same concept: to elongate the intramural segment of the ureter through submucosal tunneling. These procedures are generally considered safe and are associated with high success rates, typically between 95% and 98%, even though in high-pressure, compliance bladders, a high rate of VUR recurrence is reported when managed with ureteral reimplantation alone [7]. Among the various open surgical options, the cross-trigonal ureteral reimplantation described by Cohen remains one of the most widely used. However, a potential limitation of this method is that it may complicate future endoscopic access to the ureteral orifices as the child grows. Moreover, open surgery is associated with significant postoperative pain, hematuria, and length of stay [42,43,44].

Today, the Robotic-Assisted Laparoscopic Ureteral Reimplantation is also widely adopted, even though variable outcomes are reported. In a recent meta-analysis, the overall efficacy of this technique seems to have longer operative times, higher costs, and increased rates of secondary procedures than open surgery procedures, although it may offer advantages in terms of reduced postoperative pain and shorter recovery time [45,46]. Despite the growing interest, laparoscopic and robotic approaches are more invasive than endoscopic treatment, and their superiority over conventional open surgery remains controversial. Thus, routine use of laparoscopy for VUR correction cannot be broadly recommended; it may be considered in specialized centers with significant experience [47].

The introduction of biodegradable bulking agents has provided a minimally invasive alternative to both continuous antibiotic prophylaxis and open surgical procedures for the management of VUR in pediatric patients. This approach involves the injection of a biocompatible substance beneath the mucosal layer of the intramural ureter during cystoscopy. This injection results in reinforcement of the distal ureter, lengthening the submucosal tunnel, narrowing the ureteral lumen, and thus reducing retrograde urine flow but still preserving normal antegrade drainage [48]. Over the past three decades, various bulking agents have been used for endoscopic correction of VUR. Currently, the most widely adopted are dextranomer/hyaluronic acid (Deflux^®^) (Scandinavian Regulatory Services AB, Svärdvägen 3b, 182 33 Danderyd, Sweden)and polyacrylate-polyalkyl copolymer (PPC) hydrogel (Vantris^®^) (Via Sommariva, 35–10022 Carmagnola, Torino, Italy) [49,50,51]. Several endoscopic injection (EI) techniques have been developed over time, including the subureteral transurethral injection method, also known as the STING (Sub-ureteral Teflon Injection) procedure [52], and the hydrodistension implantation technique (HIT) [53]. In our case study, only Deflux^(R)^ was used.

Clinical outcomes are influenced by the timing of reflux observed during voiding cystourethrogram (VCUG). When reflux occurs exclusively during the voiding phase, it tends to respond more favorably to endoscopic therapy compared to filling-phase reflux, likely due to differences in bladder dynamics and pressure profiles [54].

However, several studies have demonstrated that the success rate of this approach decreases with higher grades of VUR and in neurogenic bladders. A large meta-analysis involving 5527 patients, both with neurogenic and non-neurogenic bladders, and 8101 renal units, reported reflux resolution rates of 78.5% for grade I–II, 72% for grade III, 63% for grade IV, and 51% for grade V after a single injection. Success rates declined with additional treatments (68% after a second injection, 34% after a third), with an overall cumulative success rate of 85%. Importantly, the success rate was significantly lower in neurogenic bladders (62%) compared to normal bladders (74%), despite a similar distribution of reflux grades. This suggests that bladder dysfunction, rather than reflux severity alone, contributes to treatment failure [55]. Furthermore, the complication rate is higher in neurogenic bladder, although it is very low in the general population (VUR without NBD). In a retrospective study of 745 patients, 5 reported ureteral obstruction after endoscopic injection; 4 of these had voiding dysfunction or neurogenic bladder [56]. UVJ obstruction after endoscopic treatment is usually resolved with the placement of a temporary ureteral stent, although some series report the need for ureteral reimplantation for its resolution [49].

In neurogenic bladders, thickened and poorly compliant detrusor walls, often associated with fibrosis and a high-pressure bladder, complicate the proper placement and stability of bulking agents. High detrusor pressures, if not controlled, can predispose to migration or inefficacy of the injected material. For this reason, maintaining low detrusor pressure, typically achieved with CIC and anticholinergics or BTX-A, is crucial to optimize outcomes [7].

Some authors have suggested that treating bladder dysfunction alone can improve or resolve VUR in these patients. Morioka et al. analyzed 231 patients with NBD due to spina bifida, including 151 ureters with VUR. They observed that 95% of low-grade VUR cases resolved spontaneously or after bladder augmentation without reflux correction surgery; 92% of high-grade VUR required ureteral reimplantation. Moreover, VUR recurred in 20.4% of cases treated with ureteral reimplantation alone but not in those who also underwent bladder augmentation. Therefore, urodynamic evaluation is necessary to choose the best treatment [57,58].

With the advent of intradetrusor BTX-A injections, several studies evaluated their role in improving bladder compliance and indirectly reducing VUR. Sharifiaghdas et al. evaluated 35 patients with NBD and detrusor overactivity, 32 of whom had VUR. Following BTX-A treatment, complete VUR resolution was achieved in 17 patients (53.1%), while VUR grade reduction was achieved in 13 (40.6%) [59].

Given the low-resolution rates achieved with BTX-A alone, combined approaches have been proposed. Sakalis et al. demonstrated VUR resolution in 88.9% of adult patients with spinal cord injury treated with combined Macroplastique^®^ and BTX-A, compared to 65.4% with Macroplastique^®^ alone. Additionally, combined treatment yielded better urodynamic outcomes, preventing the increase in detrusor pressure observed in the bulking agent-only group [14]. The first pediatric report of this combined management (BTX-A and Deflux^(R)^ injections) dates to 2003. Neel et al. evaluated 10 patients, all with NB due to myelomeningocele, with 16 refluxing ureters. After combined endoscopic treatment, VUR resolution was achieved in 15 ureters (93.8%) [13]. Subsequent studies confirmed superior outcomes with combined therapy compared to bulking agents alone. Mosiello et al. in a single-center retrospective study reported on seven pediatric patients (mean age 10.8 ± 8.1 years) with neurogenic bladder and VUR treated with combined intradetrusor Onabotulinum Toxin-A (10 U/kg, max 300 U) and subureteric Dx/HA injections (0.5–1 mL). All 12 renal units showed complete VUR resolution at 12–33 months follow-up, with significant urodynamic improvements. BTX-A was repeated in five patients after approximately 6.8 months. No adverse events occurred, and UTIs resolved in all patients, though two remained on antibiotic prophylaxis. In contrast, among two patients treated with BTX-A alone, VUR resolved in only one [16]. We also confirmed the safety and efficacy of TEM in this challenging pediatric population, with therapeutic success reached in 14 out of 24 ureters analyzed (58.8%).

It has been hypothesized that one of the factors that could lead to a failure of endoscopic treatment is bladder fibrosis, often found in patients with NB. In our series, 4 out of 8 non-responders presented fibrosis at histological examination. Even in the absence of statistical significance, we believe that the presence of fibrosis could make the correct positioning of the bulking agent more difficult, as well as reduce the bladder response to BTX-A. This topic has also been explored in studies evaluating the relationship between intradetrusor BTX-A injections and bladder histopathology. Temeltas et al. reported a progressive increase in bladder wall fibrosis following repeated injections, which may impair the diffusion of the toxin within the detrusor muscle, ultimately diminishing its therapeutic efficacy [60]. Conversely, Pellegrino et al. found no significant histological alterations in children after repeated injections [11].

Limits of the study: a retrospective single-center study; small patient sample; short follow-up.

## 4. Conclusions

Total endoscopic management of VUR in NBD patients is a safe and effective treatment, even for pediatric patients, with superior outcomes in terms of VUR resolution than BTX-A alone, although bladder wall fibrosis may be a factor limiting the effectiveness of treatment. An early treatment may be useful to increase TEM success, to avoid or postpone a surgical procedure.

Further studies are needed to refine patient selection and assess long-term outcomes.

This work is generated within the European Reference Network for Rare Urogenital Diseases and Complex Conditions (ERN EUROGEN).

## 5. Materials and Methods

### 5.1. Ethical Aspect

The study was approved by our Ethical Committee (number 200602R001820).

Written informed consent was obtained for all subjects involved in the study from their parents/caregivers.

### 5.2. Inclusion Criteria

We retrospectively review charts of all patients with neurogenic bladder and vesicoureteral reflux treated in our pediatric neuro-urological division from January 2020 to December 2023.

All demographic and clinical data were collected from patients’ charts.

Inclusion criteria were: TEM for symptomatic VUR in NBD patients not responsive to CIC and anticholinergic drugs, or who do not tolerate anticholinergics. We included unilateral VUR II-V grade and bilateral VUR I-V grade. Age at TEM procedure: ≥1 and <18 years. Follow-up length of at least 12 months.

Surgical indications for TEM were the presence of recurrent symptomatic UTIs, renal impairment on blood tests (serum creatinine increase), renal scarring at DMSA (dimercaptosuccinic acid), and/or renography/worsening of renal function.

We considered as “recurrent” the presence of at least 3 episodes/year or ≥2 UTIs in the last 6 months, while as “symptomatic” the manifestation of Lower Urinary Tract Symptoms (LUTS), such as dysuria, frequency, urgency, with systemic characteristics (fever, chills, flank pain) [10,61].

When borderline situations occurred (such as increased serum creatinine values but still within the normal range for age), patients were followed with a more intensive follow-up program, in order to plan timely treatment in case of clinical variations, including the criteria for surgical indication.

### 5.3. Exclusion Criteria

We excluded from the study all patients subjected to previous urological surgery, such as ureteral reimplantation or bladder augmentation.

We do not include patients not performing CIC and those with non-febrile UTIs.

Follow-up length < 12 months.

### 5.4. Patients’ Evaluation

All patients with neurogenic bladder dysfunction were firstly evaluated in our outpatient clinic, with clinical evaluation, laboratory test (comprehensive of blood and urine exams), urinary ultrasound (US) or other radiological imaging (e.g., renography, magnetic resonance) and urodynamic/videourodynamic evaluations, as recommended by the International Children’s Continence Society (ICCS) [62].

Vesicoureteral reflux was diagnosed with voiding cystourethrography (VCUG) or during videourodynamic exam. VUR severity was classified according to the International Grading System (I–V) [16]:

- Grade I: reflux is confined to a non-dilated ureter
- Grade II: reflux extends into the renal pelvis without dilation of the calyces
- Grade III: mild to moderate dilation of the ureter and renal pelvis is present, with minimal or absent blunting of the calyceal fornix
- Grade IV: moderate ureteral and pelvicalyceal dilation occurs, with obliteration of the acute angles of the calyceal fornices
- Grade V: marked tortuosity and dilation of the ureter, renal pelvis, and calyces, with loss of papillary impressions.

TEM was proposed as a second-line treatment in those patients not responding (clinically and radiologically and/or urodynamically) to first-line treatments (CIC, anticholinergic CAP).

### 5.5. TEM Protocol

**Figure 1:**
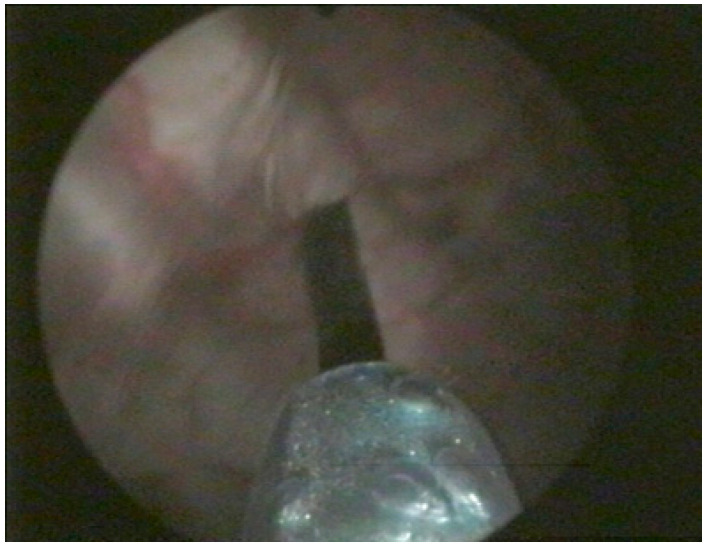
Endoscopic image of BTX-A injection.

TEM was performed during a brief hospitalization, under general anesthesia in the operating room. BTX-A injection was performed according to our protocol, previously reported [11,63,64]. All patients underwent cold-cup biopsies at the beginning of the procedure. Then BTX-A (Botox^(R)^, Botox, 100 U Allergan, AbbVie S.r.l., Campoverde di Aprilia, Latina, Italy) was injected into the detrusor (8 U/kg, maximum 200 U; 1 mL per injection, corresponding to 10 U/1 mL of saline solution), sparing the trigone, using a rigid cystoscope and flexible needle (Figure 1).

Deflux^(R)^ (Scandinavian Regulatory Services AB, Svärdvägen 3b, 182 33 Danderyd, Sweden) injection was performed right under the ureteral orifices (0.5–1 mL), according to the STING technique [52,65,66].

A bladder Foley catheter was left for 24 h after the procedure, and antibiotic therapy was administered for 5 days.

Intra- and postoperative complications, such as ureteral obstruction, hematuria, implant migration, or febrile UTI, were evaluated.

The procedure was repeated in case of failure and persistence of the patient’s clinical stability. No more than two procedures were performed over time on a single patient; in case of failure at the second TEM, the therapeutic approach was changed.

### 5.6. Follow-Up Evaluation

All patients were enrolled in our follow-up program, including at least one evaluation per year. Response of TEM was evaluated based on clinical findings (no febrile UTIs), reduction or absence of renal pelvis dilation on ultrasound exam, no VUR on subsequent videourodynamic study or VCUG, and no worsening of renal function.

### 5.7. Statistics

Data were analyzed using MedCalc^®^ Statistical Software version 20.2016 (MedCalc Software Ltd., Ostend, Belgium: https://www.medcalc.org (accessed on 3 April 2025). Results are reported as numbers with percentages or mean, specifying SD and range; *p* < 0.05 was considered statistically significant.

## Figures and Tables

**Table 1:** Patient Demographics and Clinical Characteristics.

Variable	Value
Number of pts	19
Sex distribution	10 males (52.6%) 9 females (47.4%)
Underlying diagnosis	15 spina bifida (78.9%) 4 other neurologic conditions (21.1%) *
Mean age at surgery (years)	7.6 (1.3–17.7; SD 4.8)
Bilateral VUR	5 pts (20.8%)
Total number of ureters treated	24
Prior BTX-A treatment	5 pts (20.8%)
Mean follow-up duration	3.2 years (1–4.7–SD 1.1)

Pt/pts: patient/patients. SD: standard deviation; VUR: vesicoureteral reflux; BTX-A: Onabotulinum Toxin-A. * Other neurological conditions: 2 pts central paralysis, 1 pt sequelae of sacrococcygeal teratoma and 1 pt sacral arachnoid cyst.

**Table 2:** Surgical and Postoperative Outcomes.

Variable	Value
Mean operative time (minutes)	13 (9–17; SD 2.4)
Mean hospital stay (days)	1 (1–3; SD 0.7)
Treatment success (ureters)	14/24 (58.3%)
Treatment success (patients)	11/19 (57.9%)
Persistent VUR (ureters)	10/24 (41.7%) - VUR downgrading: 6 - VUR unchanged: 4
Mean BTX-A reinjection interval	8.5 months (range: 6–14; SD 2.5)
Procedure-related complications	None

SD: standard deviation; VUR: vesicoureteral reflux; BTX-A: Onabotulinum Toxin-A.

## Data Availability

The original contributions presented in this study are included in the article. Further inquiries can be directed to the corresponding author.

## Abbreviations

The following abbreviations are used in this manuscript:

NBD	Neurogenic Bladder Dysfunction
VUR	Vesicoureteral Reflux
UTIs	Urinary Tract Infections
NB	Neurogenic Bladder
UVJ	Ureterovesical Junction
CIC	Clean Intermittent Catheterization
BTX-A	Onabotulinum Toxin-A
TRPV1	Transient Receptor Potential Vanilloid 1
P2X	P2X Purinoceptor
CAP	Continuous Antibiotic Prophylaxis
Deflux^(R)^	Dextranomer/Hyaluronic Acid
TEM	Total Endoscopic Management
Pt/pts	Patients/Patients
SD	Standard Deviation
SB	Spina Bifida
LUTD	Lower Urinary Tract Dysfunctions
NTDs	Neural Tube Defects
SCI	Spinal Cord Injury
NLUTD	Neurogenic LUTD
UUT	Upper Urinary Tracts
RIVUR	Randomized Intervention For Children With Vesicoureteral Reflux
Vantris^®^	Polyacrylate-Polyalkyl Copolymer (PPC) Hydrogel
EI	Endoscopic Injection
STING	Sub-Ureteral Teflon Injection
HIT	Hydrodistension Implantation Technique
VCUG	Voiding Cystourethrogram
LUTS	Lower Urinary Tract Symptoms
ICCS	International Children’s Continence Society
